# A Review on the Potential of Water Hyacinth to Enhance Ruminant Performance

**DOI:** 10.3390/ani16111590

**Published:** 2026-05-23

**Authors:** Khakhathi Milicent Ralinala, Thivhilaheli Richard Netshirovha, Tendani Lucky Nesengani, Ntanganedzeni Olivia Mapholi, Michael Chimonyo

**Affiliations:** 1 Department of Agriculture and Animal Health, College of Agriculture and Environmental Sciences, University of South Africa, Private Bag X6, Florida 1710, South Africa; 2 Faculty of Science, Engineering and Agriculture, University of Venda, Private Bag X5050, Thohoyandou 0950, South Africa

**Keywords:** nutritive value, processing, production performance, water hyacinth, ruminants

## Abstract

The utilization of unconventional feed resources offers a sustainable strategy to mitigate feed shortages particularly in tropical and subtropical regions where access to conventional feeds is often limited. Among these, water hyacinth (*Eichhornia crassipes*) is one of the world’s most aggressive aquatic weeds, which has drawn attention due to its dual role as a problematic invasive species and a potential livestock feed. This plant reduces water quality, contributes to biodiversity loss and causes economic damage in farming systems. At the same time, its high capacity for nutrient absorption makes it a viable source of protein and energy for ruminants when properly harvested and processed into forms such as hay, dried leaves, and silage. However, its utilization requires caution, as the plant can accumulate toxins and heavy metals from polluted water, which may harm animal health if unprocessed. This review focuses on the potential of water hyacinth to improve ruminant growth performance, nutrient digestibility and rumen fermentation. Including water hyacinth in ruminant diet safely can possibly improve animal productivity, contribute to sustainable weed management and also provide a practical strategy to alleviate feed shortage in dry seasons, thereby encouraging resilience and sustainable ruminant production.

## Simple Summary

Water hyacinth is widely available, fast-growing, and nutrient-rich, making it a promising alternative foodstuff. Studies included in the review show that moderate inclusion levels (10–30% of the diet) can support growth performance, with ruminants indicating improved daily weight gain and overall feed efficiency. Nutrient digestibility and nitrogen utilization depend on plant processing and species, with fermented or ensiled water hyacinth improving palatability and rumen utilization. While excessive inclusion may reduce intake due to its high fiber content, moderate levels enhance production without adverse effects. The review highlights water hyacinth’s role in sustainable ruminant feeding, particularly in regions with feed shortages, but further controlled trials are necessary to determine optimal inclusion rates, processing methods, and long-term impacts on animal health and productivity.

## 1. Introduction

Ruminants such as cattle, goats and sheep are vital to agriculture and global food security because of their unique digestive system. Their importance to human lives often translates beyond food security and, in many parts of the world, ruminants are well embedded within cultural and societal norms. A common challenge within ruminant farming is feed availability and quality—a limiting factor in animal production around the world. This is more expressed in the tropics, where ruminants suffer from a lack of forage and pasture quality, especially during the dry season when natural vegetation has poor nutritional value [1,2]. Thus, it is often suggested that during the dry season, non-conventional feed resources should be considered [1,3]. This often includes the use of dietary supplements and alternative feed resources, such as water hyacinth (*Eichhornia crassipes*), which has been utilized as a non-conventional feed ingredient, particularly by smallholder livestock farmers [2,4].

Water hyacinth is a wild freshwater fern belonging to the family *Pontederiaceae*. It is an erect, free-floating, stoloniferous, perennial weed that lives at the air–water interface, forming two distinct canopies: leaf canopies comprising above-water structures and root canopies comprising below-water structures [5]. The plant is native to South America but has been naturalized in many tropical and subtropical regions of the world. It grows and reproduces (by seeds and by daughter plants) at a high rate and can tolerate a wide range of environmental conditions such as temperature, humidity, light, pH, salinity, wind, current, and drought [6].

Free-floating WH plant biomass forms dense mats that block navigation and interfere with irrigation, fishing, recreation, and power generation. These mats also prevent sunlight penetration and aeration of the water, leading to oxygen deficiency, competitive exclusion of submersed plants, and reduced biological diversity. Water hyacinth contains between 300 and 600 g/kg nitrogen-free extract (NFE) and between 200 and 300 g/kg crude fiber [7]. The utilization of WH as manure to enhance mulching and soil fertility, its potential for producing animal fodder, aquafeed, and fertilizer, as well as its applications in water purification, biogas production, and even human food, has been explored in several studies [8].

Water hyacinth is available throughout the year, and considering its nutritive potential, using it as a feed alternative resource is also a way of controlling or reducing its spread. Water hyacinth, with no amendment apart from sun drying, is suitable for small-scale use, with minor nutrient losses at low cost and effort. For better results, ensiling or fermenting the plant can lead to higher animal acceptability and subsequent higher returns. Since it grows in polluted water with organic contaminants, there is a need to process it before it can be used as feed for livestock, to avoid harmful effects to animals [9]. The objective of the review is to valorize the potential of water hyacinth as an energy and fiber source for ruminants. The review focuses on water hyacinth, its uses, nutritive value, benefits, impact on animal performance, and challenges facing the use of water hyacinth as feed. Moreover, the research seeks to promote the sustainable utilization of water hyacinth in livestock farming, potentially offering an ecological remedy to its proliferation while improving feed accessibility, especially for smallholder farmers. The benefits of this review are the ability to document the potential and sustainable use of water hyacinth as a way of controlling their invasion within rivers and dams. As a result, this review will assist rural and commercial farmers, agricultural organizations, and government departments in improving ruminant production while improving the surface waters and the environment.

## 2. Methodology

This review summarizes the current scientific evidence on the utilization of water hyacinth (*Eichhornia crassipes*) as an unconventional feed for ruminant nutrition. A comprehensive search of scientific literature was carried out between January 2010 and December 2025 through electronic databases like Scopus, Web of Science, ScienceDirect, Google Scholar, PubMed, and with relevant archives such as AGRIS and FAO. The search terms included keywords such as water hyacinth, Eichhornia crassipes, ruminants, feed, nutritional value, hematology, digestibility, animal performance, and rumen fermentation. Boolean operators were used to maximize the retrieval of relevant studies.

A total of 210 studies were identified with the first search, and duplicates were removed. Titles and abstracts were screened for relevance and studies which did not include ruminants and water hyacinth or lack original empirical data were excluded. After full-text assessment, 84 articles were retained for inclusion in the review. These comprised original research articles, review articles and technical reports focusing on nutritional composition, processing methods, animal performance outcomes, hematological and biochemical responses, and anti-nutritional considerations of water hyacinth feeding.

Relevant data were extracted and organized into a standard matrix which included: author and publication year, sample size, livestock species, dietary treatments levels, hematological outcomes, serum biochemistry values, statistical significance reported from each result, and key conclusions of each study. Where possible, values were compared to recognize physiological reference ranges to provide a clinical context. Descriptive synthesis was used to interpret trends, identify consistent findings across studies, and highlight gaps in knowledge. Studies with inconsistent or insufficient reporting were noted, and recommendations for future research were developed based on observed limitations in the literature.

## 3. Ecology of Water Hyacinth

**Figure 1:**
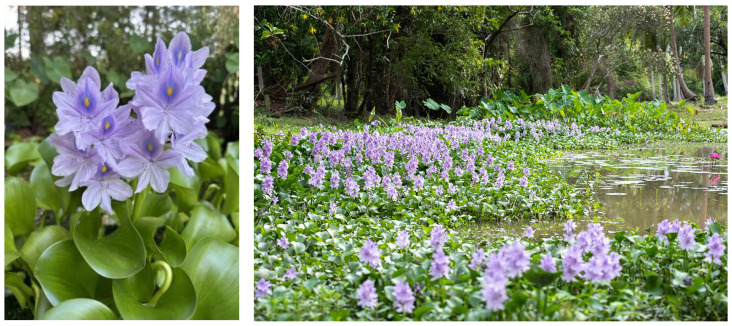
Freshwater hyacinth plants.

Water hyacinth is botanically known as *Eichhornia crassipes* (Mart.) Solms-Laubach [10] and is a member of the monocot family *Pontederiaceae* [11]. It is a freshwater plant (Figure 1) originating in the Amazon Basin and naturalized in tropical and South American subtropical countries [12]. It is recognized by its lavender flowers with six petals, and its leaves are connected to broad, shiny, thick, and oval stems, which are usually bulbous, long, and fluffy, with feathery roots [7]. The plant can tolerate both fresh and salt water [13], so that there are no aquatic boundaries to its growth and spread. It lives at the interface between air and water and forms two separate canopies—the leaf canopy develops above the water surface and the root canopy below the water surface [5]. The rate of its growth and reproduction by seed and daughter plants is very high, resulting in an increase in plant mass up to 100–400 Mt/ha per year. This forms dense mats that block navigation and interfere with irrigation, fishing, recreation, and energy production [7]. These mats also protect against sun penetration and aeration of the water resulting in reduced water oxygen levels resulting in a competitive removal of underwater plants and a reduced biodiversity [14].

Water hyacinth is considered the worst aquatic weed in the world due to its ability to form dense and impenetrable floating mats on the water surface [15]. Its invasion reduces total biodiversity in terms of richness and evenness. Factors such as temperature, availability of nutrients, salinity, light, wind, water currents, carbon dioxide levels, waves, turbidity, and water level changes can accelerate and encourage or limit and slow down infection of water hyacinths [16].

Water hyacinth (*Eichhornia crassipes*) exhibits optimal growth at water temperatures between 25 and 35 °C (optimum ≈ 28–32 °C). Its proliferation is strongly stimulated under eutrophic conditions where total nitrogen exceeds 0.5–1.0 mg L^−1^ and total phosphorus exceeds 0.05–0.10 mg L^−1^ [16,17]. Higher salinity in the water limits the reproduction and growth of water hyacinths and increases their mortality, making it difficult for coastal invasion. In cases where there is a difference between salinity and nutrients, nutrients have a greater effect on the number of leaves, while the total biomass is limited [18]. Higher levels of dissolved oxygen due to turbulence similarly limit plant growth. Water hyacinth reduces phytoplankton productivity by reducing dissolved oxygen and chlorophyll-a concentrations in water bodies covered by plants. This has a significant ecological impact on polluted water when the food web of aquatic organisms is disturbed, leading to a reduction in species composition and biodiversity of water bodies [17]. Thus, WH is an aquatic weed that harms the environment in many ways. It acts as a catalyst through sexless reproduction, demonstrates high survival adaptation and rapid growth in waters rich in nutrients such as organic matter. As it grows on the water surface and directly absorbs sunlight and oxygen, this hinders the absorption of sunlight and oxygen by aquatic organisms, resulting in a significant reduction in the dissolved oxygen content of the water. When the water is polluted and dissolved, oxygen is not timeously replenished, and anaerobic bacteria in the water body will rapidly multiply, leading to the indirect killing of microorganisms and organisms in the water [19].

In general, it is known as a notorious plant that causes severe environmental degradation and is an economic burden requiring management. However, it provides significant exploitable opportunities for communities where it is available. High temperatures, eutrophic conditions, and other environmental factors promote the proliferation of the plant. The WH root also collects a significant quantity of inorganic nitrogen and phosphate, making it an excellent source of compost or inorganic fertilizer [17]. It can be a feasible solution for feed due to its ability to absorb nutritional elements. In addition, WH can be used as a source of energy as its leaves can be anaerobically converted into biogas [19].

Nonetheless, WH is recognized as one of the world’s most aggressive and destructive aquatic invasive weeds, posing severe ecological and socio-economic challenges in infested regions [20]. Its rapid vegetative growth and adaptability make complete eradication nearly impossible without an integrated management strategy. Effective control requires a combination of biological control, chemical treatments, mechanical removal, water level regulation, and long-term community participation [11]. Sustainable management of water hyacinth can be achieved by empowering local communities and the creation of incentives through value-added utilization such as compost production, animal feed, and bio-energy generation which promote pro-environmental behavior aimed at enhancing their livelihoods [17]. Such community-based approaches not only contribute to ecological restoration but also address socio-economic challenges associated with invasive weed proliferation.

## 4. The Uses of Water Hyacinth

**Figure 2:**
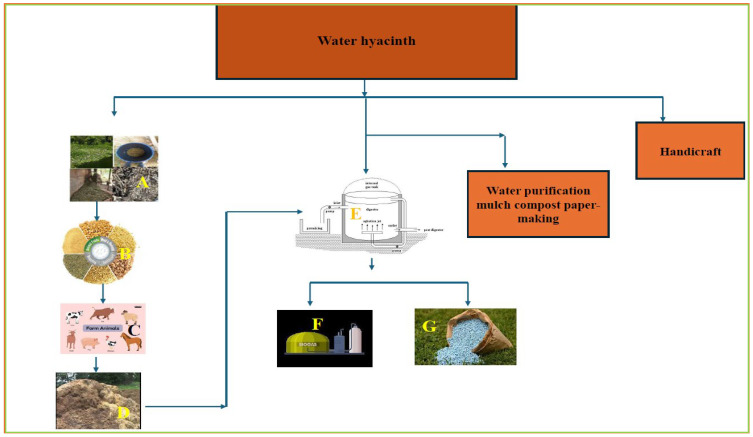
Schematic diagram of the uses of water hyacinth [32]. (**A**)—Fresh, dried, fermented, and silage; (**B**)—total mixed ration; (**C**)—livestock animals; (**D**)—animal waste; (**E**)—digester; (**F**)—biogas; (**G**)—fertilizer.

Even though water hyacinth is responsible for major environmental and economic problems, the weed has been exploited for several beneficial uses. As indicated in Figure 2, water hyacinth may be used in the production of fertilizers [19], animal feed [2,4,21,22,23], bio-energy [15,24], medicine [25], paper [26,27] and treated water [28,29,30]. Among these benefits, the nutritional potential of water hyacinth as animal feed has drawn more attention because of its high protein content, fiber composition and mineral levels [2,4]. Because of these qualities, it can be used as a feed resource, particularly in areas where traditional feed ingredients are expensive or hard to obtain. Thus, the use of water hyacinth (*Eichhornia crassipes*) as livestock feed not only promotes sustainable environmental management by controlling the spread of this invasive species but also contributes to livestock nutrition by improving feed intake, growth performance, and meat quality when included at appropriate levels in diets [21,31].

## 5. Nutritive Value

The nutritional value of feed varies significantly depending on its variety, processing methods, and source. Forage quality, including its fiber content, digestibility, and nutrient availability, is essential for ruminant health and overall productivity. Enhancing digestion and nutrient absorption is made possible by high-quality forage, which guarantees that livestock can maintain optimal health and performance levels. However, poor-quality feed can result in significant economic losses resulting from digestive problems and decreased productivity. On the other hand, feeding ruminants overly mature grasses increases the production of methane, which is strongly correlated with the carbon-to-nitrogen ratio in plants [33].

### 5.1. Proximate Composition of Water Hyacinth

The chemical composition of water hyacinth is affected by and varies with the environment in which it grows, such as the quality of the water, temperature, humidity, salinity, and pH [34]. Thus, the chemical composition of water hyacinth leaves, stems, and roots shows differences in chemical profiles. Table 1 presents the proximate composition of water hyacinth as percent DM basis.

### 5.2. Amino Acid Profile of Water Hyacinth

Amino acids are the fundamental building blocks of proteins that are crucial for multiple metabolic functions including health and immune responses, growth, development, lactation, reproduction and survival. Amino acids are referred to as essential or as non-essential. Essential amino acids such as histidine, isoleucine, leucine, lysine, methionine, phenylalanine, tryptophan, threonine and valine are amino acids that are acquired by an animal via their diet, while non-essential amino acids such as serine, glycine, alanine, proline, glutamic acid and aspartic acid are amino acids produced in the body from other amino acids or other substances [40,41].

Amino acids in ruminants primarily originate from microbial protein synthesized in the rumen and from dietary protein that escapes ruminal degradation. Microbial protein and rumen-undegradable protein are digested in the abomasum and small intestine, providing essential amino acids for absorption by the host animal [42,43]. Methionine and lysine are often the first limiting amino acids in ruminant diets, critically influencing growth, milk synthesis, and overall production performance [44]. Functional amino acids such as arginine and glutamine play important roles in immune function, reproductive health, and intestinal metabolism, beyond their contributions to protein synthesis [42,43,45]. Adequate provision of essential amino acids is vital, as deficiencies or imbalances can reduce productive performance, increase nitrogen excretion, and compromise animal health. Feed augmented with WH is a potentially rich source of amino acids and the amino acid profile of water hyacinth leaves, stems and roots is shown in Table 2.

### 5.3. Anti-Nutritional Factors of Water Hyacinth

Anti-nutritional factors (ANFs) are substances present in feed ingredients that act against the digestibility and absorption of nutrients in animal feed. Anti-nutrients limit the potential of nutrient utilization, especially proteins, vitamins, and minerals. This prevents a feed nutrient from being used to its full potential and lowers its nutritional value [47]. Available anti-nutrients in water hyacinth include tannin that binds proteins, making them less available for digestion; phytic acid that is bound to minerals such as calcium and zinc, reducing their availability; oxalates that interfere with calcium absorption and possibly cause digestive issues; and cyanide that is present in small quantities but can be toxic if consumed in high concentrations [48,49]. Fortunately, the majority of anti-nutrient compounds in plants can be eliminated by treating the plants with different processing techniques, such as drying, soaking, germination, boiling, and fermentation [50].

Adelakun et al. [51] phytochemically analyzed water lily and reported presence of many bioactive compounds including alkaloids, tannins, flavonoids, saponins and catechins. Water spinach contains more alkaloids (4.12 mg/g) compared to other aquatic plants such as water hyacinth (2.77 mg/g), water lettuce (3.86 mg/g) and (2.68 mg/g) water lily [51]. There is a need to find ways to eliminate anti-nutrients found in different feeds without compromising their nutritional value. The anti-nutritional factors present in WH and their effects are presented in Table 3.

## 6. Processing and Pre-Treatment Methods for Water Hyacinth

The use of WH as ruminant feed has several limitations. Water hyacinth contains a high fiber concentration [54], which may reduce its digestibility and voluntary intake by livestock. In addition, because the plant grows in water contaminated with organic and inorganic pollutants, it may accumulate harmful substances. Valk [9] suggested that pre-treatment is necessary before its use as livestock feed to minimize potential health risks, including toxicity, disease and nutrition imbalances. Various processing methods such as sun drying, ensiling, fermentation and supplementation with other feed ingredients can be applied to improve safety, palatability and nutritional value for livestock.

### 6.1. Effect of Sun Drying WH and Its Utilization in Ruminant Diet

Natural sun drying is a simple and affordable method that improves storage life by reducing moisture content. Freshwater hyacinth contains more than 85% water, which limits its storage life and handling characteristics. Sun drying water hyacinth improves dry matter and available concentrates of nutrients in dried biomass, thereby improving its suitability as a feed resource compared to fresh plant [4,8,55].

Feeding trials demonstrated that sun-dried WH can contribute to ruminant performance when included at appropriate levels in balanced diets. Yusuf et al. [2] reported that including 10% of sun-dried water hyacinth in the diet improved growth performance, health indicators, and economic returns in West African Dwarf goats without adverse effects. Similarly, processed water hyacinth has shown to be more acceptable to ruminants than the fresh form due to improved dry matter intake and improved palatability [8]. However, high inclusion rates of dried water hyacinth depress nutrient utilization and animal performance.

Sun-drying advantages include low processing cost, improved shelf life, reduced spoilage and moisture, improved palatability, minimal technology requirement and sustaining of the environment, while the disadvantages include possible nutrient loss during prolonged drying, limited reduction of fiber fractions, dependency on weather conditions and potential contamination risks if harvested from polluted water bodies because water hyacinth can accumulate heavy metals and other contaminants from wastewater and nutrient-rich environments [4,8]. Consequently, sun drying is an effective and accessible preservation strategy and its optimal use in ruminant feeding systems requires controlled processing conditions and appropriate dietary formulation.

### 6.2. Effect of Fermenting WH and Its Utilization in Ruminant Diet

Fermented WH provides substrates for rumen microbes, enhancing microbial balance and promoting the production of volatile fatty acids (VFAs), which are essential energy sources for ruminants [56,57]. Fermentation also helps reduce anti-nutritional factors and pathogenic microbes, making the feed safer and more digestible [56].

Research has demonstrated that fermentation of WH using fungal or bacterial inoculants can produce a higher-protein, more digestible feed. Suharman et al. [58] reported that fermentation of WH with *Aspergillus niger* increased crude protein content to approximately 185 g/kg dry matter. Other studies have explored the use of mixed microbial cultures, including yeasts and lactic acid bacteria, to improve the fermentation quality and nutrient retention of WH-based feeds [59,60].

Fermented feeds typically exhibit higher nutrient concentrations, improved fiber digestibility, and enhanced palatability, which in turn improve feed intake, nutrient absorption, and animal performance compared to non-fermented forages [21].

### 6.3. Effect of Ensiling Water Hyacinth and Its Utilization in Ruminant Diet

Ensiling WH (*Eichhornia crassipes*) is important for ruminant feeding and environmental management because it converts a fast-growing, nutrient-rich aquatic weed into a stable, nutritious, and palatable feed. The process preserves the forage for extended periods, which is crucial during dry seasons or periods of forage scarcity, ensuring a consistent feed supply [61]. Ensiled WH has a higher dry matter (DM) and crude protein (CP) content and a lower fiber content than fresh material, improving digestibility and rumen utilization, which supports better growth and productivity in ruminants [62]. The use of additives such as molasses, rice bran, or rice straw further enhances nutrient content and palatability, encouraging voluntary intake. Additionally, replacing conventional roughages with WH silage can reduce feeding costs, divert a problematic weed from waterways, and in some cases lower enteric methane emissions, contributing to environmental sustainability [63]. Overall, ensiling WH provides a practical, economical, and eco-friendly strategy for ruminant nutrition, balancing both animal performance and resource management.

Feeding trials with ensiled WH can improve nutrient digestibility and intake in ruminants. In crossbred cattle, replacing 15–45% of rice straw in diets with ensiled WH improved crude protein, organic matter, neutral detergent fiber and acid detergent fiber digestibility, suggesting that ensiled WH can enhance feed utilization when integrated into roughage-based diets [64]. In sheep, Nguyen [65] found that replacing 30% of para grass with ensiled WH had no adverse effect on rumen characteristics but improved growth performance and feed utilization. Similarly, Yosef [66] observed that partial replacement of conventional forages with ensiled water hyacinth had a positive effect on total digestible nutrients and a significant effect on digestible energy and metabolic energy, indicating improved energy availability. In goats, Trung et al. [63] reported that replacing traditional forage with ensiled water hyacinth improves daily weight gain and feed efficiency and may reduce enteric methane emissions, suggesting potential environmental benefits alongside nutritive value. Collectively, these results suggest that ensiling water hyacinth can make a valuable supplemental roughage for ruminants, particularly in areas with limited forage availability. Additionally, controlled performance studies are required to refine inclusion levels, processing methods, and long-term effects on animal productivity and health.

## 7. Effect of Water Hyacinth When Fed to Ruminants

The use of water hyacinth (*Eichhornia crassipes*) as a feed resource in ruminant diets has drawn increasing interest due to its wide availability, high biomass yield, and acceptable nutrient profile when properly processed. Though traditionally regarded as an invasive aquatic weed, water hyacinth has demonstrated potential as a cost-effective feed supplement for various ruminant species, including goats, sheep, cattle, and calves. The effect of water hyacinth on ruminants when included in their diet encompasses improvements in the following: growth performance of ruminants, nutrient digestibility, nitrogen utilization, rumen fermentation, blood metabolites, reproduction performance, milk production, carcass characteristics and meat production [23,57,67,68].

### 7.1. Growth Performance of Ruminants

The growth performance in ruminants has been evaluated, with evidence suggesting the improvement in feed utilization and weight gain at specific inclusion levels. Fitrihidajati and Isnawati [69] reported that fermented WH has the potential to increase weight gain in goats by 2.07 kg per month. Similarly, feeding West African Dwarf goats with 10% inclusion of WH in the diets results in growth performance similar to the control group, with a body weight gain of 21.26 g/day and a final body weight gain of 8.29 kg [2].

In sheep, Isnawati et al. [23] reported that 20% fermented WH combined with corncob showed the highest weight gain (5.96 kg) in male sheep. Contradicting results were found on Amit [70] reporting that Napier grass supplemented with 20% water hyacinth for 14 weeks showed no improvement on body weight gained. Additionally, fermented WH with palm fronds and sago stems improved average feed consumption (5.46 kg/day), feed efficiency (6.23%), and average weight gain (43.92 kg) in young Acehnese cattle [71].

Although these observations suggested that WH inclusion can improve growth performance in ruminants, further studies are required to determine inclusion levels, supplementation strategies, and replacement ratios of WH across different production systems.

### 7.2. Nutrient Digestibility

Digestibility of nutrients in ruminants refers to the proportion of feed that is broken down, absorbed, and utilized by the animal. This process is influenced by dietary composition, feed processing, environmental conditions, and animal-related factors such as species and age. Because digestibility directly affects nutrient availability and animal productivity, evaluating the digestibility of unconventional feeds is essential before their practical application [72].

Water hyacinth has been offered to ruminants as fresh forage, hay, or silage. Dong and Van Thu [67] reported differences in digestibility among diets containing 25%, 50%, and 75% fresh WH fed with rice straw and multi-nutrient cake to local yellow cattle, with 50% inclusion yielding the highest digestibility. However, these values were lower than those observed by Dong and Van Thu [22] in swamp buffaloes fed 50% fresh WH with para grass and urea–molasses cake, likely reflecting species differences in rumen capacity and adaptation to fibrous aquatic plants.

Fanta et al. [73] observed higher nutrient digestibility in sheep than in goats when WH was replaced with commercial concentrate, whereas Mekuriaw et al. [74] reported greater digestibility in sheep fed 50% wilted WH with concentrate. Nguyen [65] found lower digestibility values in sheep fed 15% ensiled WH with para grass. Overall, variation in digestibility across studies may be attributed to differences in inclusion level, processing method, basal diet, and species-specific digestive capacity. Table 4 shows the total tract apparent digestibility of ruminants fed on WH-based diets.

### 7.3. Nitrogen Utilization

Nitrogen intake and retention are key indicators of protein utilization when evaluating WH as a feed resource for ruminants. Reported values vary depending on processing method, inclusion level, and animal species. In sheep, Nguyen [65] reported nitrogen intake of 1.81 to 7.94 g/day and retention of 0.639 to 0.809 g/kg when ensiled WH partially replaced para grass. Compared with the control diet of para grass only, nitrogen retention was maintained and slightly enhanced at moderate inclusion levels, suggesting no negative effect on the utilization of protein.

In West African Dwarf goats, ensiled WH combined with breadfruit resulted in nitrogen intake of 2.47–3.39 g/day and retention of 0.09–0.12 g/kg, which was lower than the control diets with no WH inclusion [75]. Similarly, Mako and Ikusika [57] observed nitrogen intake of 6.4–7.02 g/day and retention of 0.10–0.107 g/kg in goats fed fungal-treated WH, and the values were comparable to the control treatments.

Higher nitrogen intake (14.3–14.9 g/day) and retention (0.58–0.76 g/kg) were reported in Boer crossbred goats when ensiled WH replaced elephant grass. These findings suggest that WH can support moderate nitrogen utilization in ruminants, although responses depend strongly on treatment method and dietary inclusion level. However, limited data are available on nitrogen balance parameters, indicating the need for further controlled studies to clarify the protein value of WH in ruminant feeding systems.

### 7.4. Rumen Fermentation

Rumen fermentation is affected by WH inclusion and treatment. Previous studies indicate that ensiled or treated WH maintains rumen pH within the normal range and supports ammonia–nitrogen levels sufficient for microbial protein synthesis [57,65]. However, excessive inclusion of untreated WH may lead to lower fiber digestibility and altered volatile fatty acid profiles.

Water hyacinth, when properly processed, can be included in ruminant diets without adversely affecting rumen fermentation. Tumambing et al. [68] reported that WH can be fed to ruminants without any effect on rumen metabolism. Mako and Ikusika [57] reported that fungal-treated water hyacinth improves ruminal fluid parameters, which was in line with study results reported by Nguyen [65] as to the effects of WH silage in diets on feed intake, digestibility and rumen parameters of sheep. This author concluded that ensiled WH can be used to feed growing sheep without adverse effects on rumen parameters. Dong and Van Thu [67] also reported that the rumen environment of cattle supplemented with fresh WH is good for the rumen microbial activities. Table 5 shows the ruminal characteristics of ruminants fed on WH-based diets.

### 7.5. Blood Metabolites of Ruminants

Feeding and production systems influence hematological and serum biochemical variables in ruminants. However, limited studies have evaluated the effects of WH supplementation on hematological and serum biochemical parameters in ruminants. The available evidence suggested that WH inclusion may influence blood indices associated with nutritional status and physiological function.

Despite these findings, studies on the effects of WH on the blood metabolites of cattle are lacking, with only limited studies available on sheep and goats. Further research is necessary to bridge this knowledge gap and understand the effect of alternative feed resources on the blood profiles of ruminants. Table 6 shows the few studies that have evaluated the effects of feeding WH at different inclusion levels on ruminant blood profiles.

### 7.6. Reproduction Performance in Male Ruminants

Under-nutrition primarily affects male reproductive performance in ruminants, primarily through reduced semen quality and impaired spermatogenesis. Nutritional status plays a vital role in determining spermatozoa concentration, morphology, motility and reproductive efficiency. Adequate dietary feed intake has been associated with reduced sperm abnormalities and improved semen characteristics [77].

Isnawati et al. [23] reported that inclusion of fermented WH at 30% in the diet of rams significantly improved reproductive performance, producing spermatozoa of superior quality characterized by increased ejaculate volume (1.05 mL) and high mass motility. These findings align with the research published by Ratnasari et al. [78] who observed that inclusion of fermented WH in feed at 35% improved spermatozoa quality for goats compared to conventional feeding regimes. These findings suggest that fermented WH supplementation can positively influence semen quality in male ruminants. However, there is limited information on the effect of WH supplementation on reproductive performance in both female and male ruminants. Therefore, further studies are necessary to evaluate its potential impact on reproductive outcomes of both sexes.

### 7.7. Milk Production

Improving the production of high-quality dairy and livestock products is one of the most significant strategies for advancing animal husbandry worldwide [79]. However, there is limited information regarding the impact of WH on milk yield and quality in ruminants. Understanding the effect of WH supplementation is essential to determine its suitability in dairy production system.

Tumambing et al. [68] reported that dairy cows fed a diet supplemented with 40% WH and banana pseudostem improved milk yield, with average daily production ranging between 9.09 and 9.33 L per day. The study also reported favorable changes in milk composition, including milk fat (3.21–4.39%), solids-not-fat (8.22–8.82%), total solids (11.83–12.98%), protein (3.12–3.33%), and sugar content (1.39–1.79 mg/mL), without adverse effects on animal performance. Despite these promising results, this remains the only available study conducted on the effect of WH supplementation in relation to milk production. Therefore, further research is required to evaluate different inclusion levels of WH and their effect on milk yield and quality in ruminants.

### 7.8. Carcass Characteristics and Meat Production

Few studies have investigated the effect of WH supplementation on carcass characteristics and meat quality in ruminants. Available studies suggested that WH inclusion in diets may influence carcass and muscle development.

Mani et al. [80] reported that dietary inclusion of 10% WH improved carcass characteristics in Awassi sheep, resulting in higher live weight (33.05 kg), hot carcass dressing percentage (50.90%), longissimus dorsi length (15.73 cm) and eye muscle area (404.1 mm), indicating improved meat yield. Similarly, Fitrihidajati and Isnawati [69] reported that goats fed 35% WH combined with dried green kangkong and solid tofu dregs (feed formula III) produced meat with crude protein (18.8%), crude fat (2.28%), ash (1.06%), calcium (2.32%), metabolize energy (977.78 kcal/kg), with an increase in meat protein of 1–1.5%. The observation suggested that WH supplementation may positively affect carcass yield and meat nutritional composition. However, further research is required to confirm the effects and inclusion levels across different ruminant species. The effect of WH on the performance of livestock is shown in Table 7.

## 8. Challenges and Feeding Strategies of Using Water Hyacinth as Feed for Ruminants

The use of water hyacinth (*Eichhornia crassipes*) as a feed resource for ruminants presents both challenges and nutritional opportunities which must be addressed through appropriate feeding strategies. When fed alone for extended periods, fresh WH cannot maintain body weight and may result in malnutrition and metabolic disorders [73]. Therefore, WH should not solely be used as feed but rather as a supplemental component within balanced diets.

The major concern in WH utilization is its ability to absorb heavy metals and environmental pollutants such as lead, chromium, arsenic, mercury, and fluorine from contaminated water bodies [81]. The roots accumulate up to three times more toxic elements than the aerial parts [82]. However, harvesting WH from an uncontaminated water source, removing the root before feeding and screening for periodic heavy metals are strategies that can assist in the safe feeding of WH.

WH is bulky and high in crude fiber in ruminants, reducing digestibility and voluntary intake when fed alone. Additionally, anti-nutritional factors such as hydrolysable tannins and oxalates may interfere with nutrient utilization [83]. However, previous studies indicated that moderate inclusion levels (10–30%) improve growth performance of ruminants. Research should also clarify the mechanisms influencing nutrient digestibility, which varies with species, processing method, and inclusion level [2,69,70,71,84]. This includes evaluating the roles of anti-nutritional factor reduction, microbial adaptation, and fermentation characteristics.

Studies have shown that properly processed WH does not affect rumen fermentation and may improve nitrogen retention with ensiled or treated WH [57,65,75]. However, there is limited data on rumen microbial adaptation and ammonia dynamics, and it requires future investigation.

Major gaps remain in animal health and production. Limited data are available regarding the effect of WH on blood metabolite responses, milk production, reproductive indices, and carcass quality. Comprehensive physiological assessment is lacking and requires investigation.

## 9. Conclusions

In conclusion, water hyacinth can serve as a supplementary feed resource for ruminants when properly processed and incorporated at moderate inclusion levels. The findings support the strategic use of WH as a locally available and low-cost feed ingredient, particularly when resources are limited. This research observed that the inclusion of WH in balanced rations supports growth performance without any negative effects on animal health. Processing methods such as drying, fermentation or ensiling improve nutritional value and intake, while dietary combination with other conventional feed ingredients improves overall nutrient utilization.

Further studies are required to determine optimal inclusion levels, long-term health and production effects, and assess the potential contaminant transfer into animal products. Appropriate processing and dietary balancing of WH can be integrated into ruminant feeding systems as an economically and environmentally viable alternative feed resource.

## Figures and Tables

**Table 1:** Proximate composition of water hyacinth as percent DM basis.

By-Product	CP	CF	EE	Ash	Carbohydrates	DM	References
Fermented leaves & stalks	18.35	-	4.86	12.26	62.36	81	[35]
Dried leaves	21.44	37.66	0.23	11.11	17.86	-	[36]
Dried roots	6.86	24.47	0.60	59.27	3.67	-	[36]
Dried whole plant	10.5	22.66	11.48	9.75	-	85.34	[2]
Fermented whole plant	14.56	25.91	4.35	19.12	-	21.5	[37]
Silage leaves	12.34	-	-	14.64	-	-	[22]
Dried whole	13.73	9.60	2.37	6.63	-	7.33	[38]
Whole plant fraction	8.2	21.6	-	18.2	49.98	89.20	[39]

Abbreviations: DM, dry matter; CP, crude protein; CF, crude fiber; EE, ether exact; %, percentage.

**Table 2:** Amino acid profile (mg/100 g dry matter) of the leaf, stem and root of water hyacinth.

Amino Acid (mg/100 g)	Leaf	Stem	Roots	Leaf
Glycine	4.67	6.07	4.84	6.51
Alanine	6.98	6.77	6.82	6.45
Serine	4.20	4.25	3.50	10.21
Proline	2.68	2.40	2.57	5.62
Valine	3.36	3.09	2.95	7.46
Threonine	4.38	3.22	3.52	5.27
Isoleucine	3.06	4.58	2.94	5.47
Leucine	7.02	8.08	8.69	9.56
Asparagine	8.40	7.59	8.19	10.21
Lysine	7.73	5.21	6.06	5.06
Methionine	2.09	1.33	1.47	1.31
Glutamine	15.13	11.95	15.12	7.31
Phenylalanine	4.29	4.53	3.91	6.01
Histidine	2.93	2.01	3.01	2.22
Arginine	5.25	8.06	4.40	6.58
Tyrosine	2.20	2.33	2.10	2.92
Cysteine	1.78	2.31	2.18	0.38
References	[36]	[36]	[36]	[46]

**Table 3:** Anti-nutritional factors present in water hyacinth and their effects.

ANF	Effect	Levels/Concentrations	Plant Part	References
Oxalates	Bind calcium and magnesium; may cause kidney stones and reduce mineral bio-availability	2–5% DM	Leaves, whole plant	[47]
Tannins	Reduce protein digestibility by binding proteins and inhibiting digestive enzymes	0.56 mg/100 g DM	Whole plant	[39]
Phytates	Bind essential minerals such as iron, zinc, calcium to reduce their absorption	0.2–2% DM	Leaves, whole plant	[52]
Saponins	Interfere with nutrient absorption and may have toxic effects at high levels	<1% DM	Leaves, stem	[53]
Alkaloids	Some may be toxic or interfere with metabolism	<0.5% DM	Whole plant	[47]
Cyanide	Can be toxic if consumed in high concentrations	1–12 mg/kg DM	Whole plant	[48,49]

Abbreviation: ANF—anti-nutritional factor, DM, dry matter; values are expressed on a dry matter (DM) basis unless stated otherwise. Ruminants can partially detoxify certain compounds (e.g., phytate, cyanogenic glycosides) through rumen microbial activity. Levels may vary with plant maturity, season, and water source.

**Table 4:** In vivo apparent nutrient digestibility of ruminants fed on processed WH.

Species	WH Processing	WH Inclusion Level on Diet (%)	CP (%)	OM (%)	DM (%)	NDF (%)	References
Sheep	Silage	45.0	78.3	67.3	66.8	66.1	[65]
Sheep	Wilted	50.0	79.2	73.2	72.8	71.8	[74]
Cattle	Freshly cut	50.0	67.3	65.4	63	64.3	[67]
Buffaloes	Freshly cut	50.0	71.6	73.4	72.4	72.7	[22]

Abbreviations: WH, water hyacinth, CP, crude protein; OM, organic matter; DM, dry matter; NDF, neutral detergent fiber.

**Table 5:** In vivo, total rumen fluid characteristics of ruminants fed with WH.

			Ruminal Parameters			
Animal	WH-Type	Inclusion (%)	pH	N-NH_3_ (mg/100 g)	VFAs (mmol)	References
Goats	Silage	25, 50 & 75	7.05–7.50	23.1–27.7	73.5–85.2	[63]
Cattle	Fresh	0, 25, 50 & 75	7.03–7.10	20.8–22.8	84.6–91.0	[67]
Sheep	Silage	0, 15, 30 & 45	6.87–7.08	34.6–38.5	108–115	[65]
Goats	Fungal-treated	0, 30, 45, 60 & 90	6.15–6.31	20.1–25.6	55.2–76.1	[57]
Buffaloes	Fresh	25, 50, 75 & 100	6.95–7.05	17.5–25.2	74.2–87.2	[22]

%, percentage; N-NH_3_, ammonia nitrogen; VFAs, volatile fatty acids; mg, milligram; g, gram; mmol, millimole; WH, water hyacinth.

**Table 6:** Effects of WH inclusion in diets on hematological and serum biochemical parameters of ruminants.

Species	WH Inclusion	Parameters Reported	Notable Means (Units)	Reference
Goat	0%, 5%, 10%, 15%	PCV, RBC, Hb, WBC, albumin, globulin, ALT, AST, TP, cholesterol, creatinine	PCV: 27.75–36.25%; Hb: 93–120.8 g/L; RBC: 4.68–6.08 × 10^12^/L; albumin: 32.6–37.8 g/L; globulin: 26.3–32.2 g/L; AST: 27.4–46.3 U/L	[2]
Goat	WH in cassava peel + poultry droppings silage (15% WH)	WBC, RBC, Hb, PCV, total protein, albumin, globulin, glucose, creatinine	WBC: 9.78–13.48 × 10^9^/L; RBC: 7.75–13.82 × 10^12^/L; PCV: 24.4–29.73%; TP: 20.02–42.24 g/L; albumin: 11.4–25.0 g/L; globulin: 8.62–17.24 g/L; creatinine: 1.00–2.13 mg/dL	[76]
Sheep & Goat	0–75% WH replacing concentrate	AST, ALP, ALT, albumin, globulin, GLU, creatinine, UREA	Sheep had higher AST, ALP, globulin; goats had higher albumin	[73]
Sheep & Goat	0–75% WH in hay/concentrate diet	WBC, Hb, RBC, PCV, MCH, MCHC, RDW	Sheep showed higher WBC, Hb, RBC, PCV; goats showed higher MCH, MCHC	[21]

TP, Total Protein; ALT, Alanine Aminotransferase; AST, Aspartate Aminotransferase; ALP, Alkaline Phosphatase; GLU, Glucose; PCV, Packed Cell Volume; RBC, Red Blood Cell Count; WBC, White Blood Cell Count; Hb, Hemoglobin; MCH, Mean Corpuscular Hemoglobin; MCHC, Mean Corpuscular Hemoglobin Concentration; RDW, Red Cell Distribution Width.

**Table 7:** Effect of processed water hyacinth meal on ruminant performance.

Species	Inclusions Levels (%)	Processing	Response	References
Sheep	0, 50, 75 & 100	Wilted	Wilted WH leaves substitute concentrate mix up to 75% results in the optimum growth of Washera sheep.	[74]
Sheep	0, 10, 20 & 30	Fermented	20% fermented WH and corncob have the highest palatability and WG in male sheep. Sheep fed 30% fermented WH and corncob produced spermatozoa with the highest quality.	[23]
Awassi Lamb	0, 5, 10 & 20	Fodder	WH leaf substitute concentrate mix up to 10% has optimum growth of Awassi sheep.	[80]
Dwarf goats	0, 15, 30, 45 & 60	Silage	The optimal level of WH supplementation for West African Dwarf goats was determined to be 15%, as this level resulted in the best hematology and biochemical indices, with no negative impact on their blood indices.	[76]
West African Dwarf goats	0, 5, 10 & 15	Sun-dried	Feeding West African Dwarf goats with WH up to 10% in their diets had favorable effects on growth performance, health, and possibly immune response as well as profitability.	[2]
Goats	0, 50, 60, 70, 80 & 90	Chopped and Silage	60% WH diets ensiled with 30% breadfruit improved DWG and FCR.	[75]
Swamp buffaloes	0, 25, 50, 75 & 100	Silage	WH can replace para grass in the buffalo diet up to 100%. 50% of WH replacement improves utilization of nutrients and energy, lowers feed cost, and improves the environment.	[22]
Dairy bull calves	0, 10, 20 & 40		40% WH inclusion increased intake in calves but did not have any effect on the BWG of the calves. A WH inclusion rate of 10–20% DM basis can substitute for Napier grass.	[70]
Local yellow cattle	0, 25, 50 & 75	Fresh	Fresh WH improves dietary nutrient digestibility, metabolizable energy, and positive live weight change. The optimum level of WH replacement to rice straw in cattle diet is 50%.	[67]

Abbreviations: WH, water hyacinth; %, percentage; DWG, daily weight gain; BWG, body weight gain; FCR, feed conversional ration; WG, weight gain.

## Data Availability

No new data were created or analyzed in this study. Data sharing is not applicable.
